# Development, survival, and feeding behavior of *Helicoverpa zea* (Lepidoptera: Noctuidae) relative to Bt protein concentrations in corn ear tissues

**DOI:** 10.1371/journal.pone.0221343

**Published:** 2019-08-19

**Authors:** Tom R. Bilbo, Francis P. F. Reay-Jones, Dominic D. Reisig, Jeremy K. Greene, Matthew W. Turnbull

**Affiliations:** 1 Clemson University, Department of Plant and Environmental Sciences, Pee Dee Research and Education Center, Florence, South Carolina, United States of America; 2 North Carolina State University, Department of Entomology and Plant Pathology, Vernon G. James Research and Extension Center, Plymouth, North Carolina, United States of America; 3 Clemson University, Department of Plant and Environmental Sciences, Edisto Research and Education Center, Blackville, South Carolina, United States of America; 4 Clemson University, Department of Plant and Environmental Sciences, Department of Biological Sciences, Clemson, South Carolina, United States of America; Nigde Omer Halisdemir University, TURKEY

## Abstract

The corn earworm, *Helicoverpa zea* (Boddie) (Lepidoptera: Noctuidae), preferentially oviposits and feeds on ears of corn (*Zea mays* L.) and can be managed using transgenic hybrids that produce insecticidal proteins from the bacterium *Bacillus thuringiensis* (Bt). Concentrations of Bt proteins can vary spatially and temporally in plant tissues, creating a heterogeneous environment that can increase the risk of resistance development. We planted small-plot trials of nine Bt and non-Bt corn hybrids in South Carolina in 2016 and 2017 and investigated the development, survival, feeding injury, and feeding behavior in corn ear tissues. ELISA was used to quantify the concentrations of Cry1F and Cry2Ab2 in young silk, old silk, maternal tip tissue, kernels, and husk. Cry1F and Cry2Ab2 significantly varied with silk age and both proteins were generally highest in the silk and tip tissue. Hybrids with pyramided proteins significantly reduced feeding injury to the silk, tip, and kernel ear tissues, which was less apparent with single Bt protein hybrids. The pyramided hybrid expressing Vip3A incurred no injury to either the ear tip or kernels, and only eight 1^st^ instar larvae were collected in the silk of 520 sampled ears. Age of larvae significantly varied among ear tissues but not between hybrids. Depending on hybrid family, mean larval instar in the silk, tip, and kernels was 1^st^ or 2^nd^, 3^rd^, and 5^th^, respectively. Instar-specific feeding penetrance into corn ears increased with age but did not differ between hybrids. We characterized the instar- and tissue-specific feeding behavior of *H*. *zea* larvae but did not detect differences in feeding behavior between Bt and non-Bt hybrids. Implications for resistance management strategies such as seed mixtures are discussed.

## Introduction

The corn earworm, *Helicoverpa zea* (Boddie) (Lepidoptera: Noctuidae), is a highly polyphagous insect native to North and South America that is a major pest to a number of agricultural crops [1]. Since 1996, *H*. *zea* is one of several insect pests that has been managed in corn (*Zea mays* L.) and cotton (*Gossypium* spp.) using transgenic hybrids expressing insecticidal proteins from the bacterium *Bacillus thuringiensis* (Bt) [2]. The planting of Bt crops has resulted in a number of operational, environmental and ecological benefits including reduced insecticide use, reduced impact on non-target invertebrates, protection of yield, farm-level cost savings, and widespread suppression of some insect pests [3–9]. Because of these benefits, the adoption of Bt crops has increased dramatically and more than 80% of all corn and cotton planted in the United States in 2018 expressed one or more Bt proteins [10]. However, this increased adoption equates to increased selection pressure, and the greatest threat to the durability of Bt crops is the evolution of resistance [11, 12]. Recent studies have reported the widespread resistance of *H*. *zea* to several Bt proteins in the United States [13–16].

*Helicoverpa zea* preferentially oviposits and feeds in reproductive structures of plants, such as corn ears [1], where oviposition on silks occurs from the silking stage (R1) until as late as the dough stage (R4) [17]. After eclosion, larvae feed on silk tissue before moving into the ear to feed on the ear tip and kernels, whereby younger larvae commonly graze on tip kernels before maturing and consuming whole kernels [17–19]. After larval feeding is complete, larvae exit the ear, drop to the ground, and burrow into the soil to pupate.

In Bt corn hybrids, concentrations of Bt toxins can vary by tissue type, tissue age, and environmental condition [20, 21], exposing larvae to a heterogeneous distribution of toxins in both space and time. While the silk and tip are maternal F_1_ tissue, kernels largely consist of F_2_ endosperm (although the surrounding pericarp is F_1_). Depending on the parental lines for an F_1_ hybrid, not all F_2_ kernels may express Bt toxins or express toxins at the same concentration [22, 23]. Furthermore, to delay the development of resistance, seed mixtures (“refuge-in-a-bag”) of Bt and non-Bt hybrid seeds have been proposed as one strategy to provide a refuge for susceptible insects and/or to ensure refuge compliance [24]. However, for ear-feeding insects, non-Bt ears in seed mixtures will be pollinated by Bt plants and express Bt toxins in some proportion of the kernels [22].

The consequences of *H*. *zea* feeding in corn ears with heterogeneous Bt toxin expression are that it can increase the dominance of resistance and provide the opportunity for behavioral responses [25–27]. Aversion to Bt toxins has been demonstrated in laboratory experiments with *H*. *zea* [28, 29]. In field experiments, altered feeding patterns of *H*. *zea* in ears expressing Cry1Ab have been reported. Dowd [30] reported that the occurrence of “railroading” (larvae feeding down silk channels, slightly damaging but not consuming kernels) was more prevalent in hybrids with high Bt concentrations than hybrids with low or no Cry1Ab concentrations. Horner et al. [31] analyzed the spatial pattern of kernel feeding and found that feeding injury in non-Bt ears was generally compact while feeding in Bt ears produced “scattered, discontinuous patches of partially consumed kernels, which were arranged more linearly.” Mathematical modelling has suggested that larval movement in heterogeneous ears of Bt corn significantly influences the evolution of resistance [19].

No study has provided a comprehensive characterization of where and when each larval instar is feeding and how feeding behavior among the three main tissues (silk, tip, and kernel) is influenced by Bt toxin concentrations in corn. Previous studies on ear feeding behavior of *H*. *zea* also do not include complementary data quantifying Bt toxin concentration in the tissues being consumed. The goal of this study was to test the null hypothesis that *H*. *zea* instar- and tissue-specific feeding behavior is the same between Bt and non-Bt corn hybrids. We characterized the effects of Bt and non-Bt corn hybrids on larval survival, development, and feeding behavior in the field and recorded when and where individual larvae were feeding in each corn hybrid. Additionally, we quantified the concentration of Cry2Ab2 and Cry1F in different ear tissues at different times to better understand the factors influencing larval movement on ears and to assess how feeding behavior on ears impacts the risk of resistance development.

## Materials and methods

### Ethics statement

No protected species were involved in the study. Study was conducted at the Clemson University Pee Dee Research and Education Center for which the authors had permission to access and collect data.

### Field trial design

Field studies were conducted in 2016 and 2017 at the Clemson University Pee Dee Research and Education Center in Florence, SC. A randomized complete block design with four replications was used with corn hybrid as the fixed effect. Transgenic field corn hybrids expressing Bt toxins as well as their non-Bt near isolines were obtained from Monsanto Company (St. Louis, MO) and Dow AgroSciences (Indianapolis, IN) (Table 1). For the purpose of this study, families of non-Bt and Bt near isolines that share the same background germplasm were grouped into three families (Table 1). Corn was planted in 8-row plots by 15.2 m in length. Planting dates were 19 April 2016 and 13 April 2017 and within the recommended planting window for South Carolina. Plant populations, fertilizer, and herbicide applications were used according to recommended Extension guidelines. Corn was irrigated as needed and Bt plant identity was verified for each plot using ELISA strips (Envirologix Inc., Portland, ME).

**Table 1:** Corn hybrids planted in Florence, South Carolina, 2016–2017.

Trade Name	Hybrid	Family^a^	Bt Event	Bt Protein(s)
Non-Bt	DKC64-27	DK1	—	—
YieldGard VT Triple	DKC64-24	DK1	MON810	Cry1Ab
Non-Bt	DKC64-82	DK2	—	—
Genuity VT Double PRO	DKC64-89	DK2	MON89034, MON88017	Cry1A.105 + Cry2Ab2
Genuity SmartStax	DKC64-87	DK2	MON89034, MON88017, TC1507	Cry1A.105 + Cry2Ab2 + Cry1F
Non-Bt	P1319R	Pioneer	—	—
Herculex I	P1319HR	Pioneer	TC1507	Cry1F
Optimum Intrasect	P1319YHR	Pioneer	TC1507, MON810	Cry1F + Cry1Ab
Optimum Leptra	P1319VYHR	Pioneer	TC1507, MON810, MIR162	Cry1F + Cry1Ab + Vip3Aa20

^a^Family indicates grouping of near isolines of Bt and non-Bt hybrids. Family DK1 not planted in 2017.

### Feeding injury in corn ear tissues

Sampling of corn ears in 2016 for *H*. *zea* larvae and feeding injury was initiated when ears were at the blister stage (R2) and silks had started browning. Sampling of corn ears in 2017 was initiated when approximately 50% of the ears in a plot were silking (R1). Sampling was conducted weekly until kernels dried and larvae were no longer present. Sampling dates in 2016 were 28 June, 7, 12, 19, 26 July, and 2 and 9 August. Sampling dates in 2017 were 20, 27 June, 5, 11, 18, and 25 July. Each week, 10 ears from each plot were randomly harvested at least 1.5 m from either end of rows 4 and 5 and rated for injury to silk tissue, tip tissue, and kernels. Here, we define tip tissue as the maternally-derived tissues located in the ~3 cm end of the ear, distal to the ear shank as defined by Horner et al. [31] and Caprio et al. [19]. This includes unfertilized corn ovules but not fertilized kernels. Silk injury was rated as both an estimation of the number of silk strands injured by *H*. *zea* and an estimation of the overall proportion of silks injured on each ear. Silk injury was recorded for only the first four and five weeks in 2016 and 2017, respectively, due to drying down. Tip and kernel feeding were measured as area (cm^2^) injured.

### Sampling of *H*. *zea*

Each sampled ear (described above) was carefully examined for the presence of *H*. *zea* larvae on the silk, tip, or kernel tissue. All living larvae were collected and frozen at -20°C to halt development. Larvae were counted per ear and measured individually for body length (cm), body mass (mg), and head capsule width (mm). Instar was determined using head capsule width limits for each instar[32]. Additionally, a frequency distribution histogram using all sampled larvae confirmed head capsule limits and also that there were six instars defined here as: 1^st^ = 0.20–0.39 mm, 2^nd^ = 0.40–0.50 mm, 3^rd^ = 0.51–1.00 mm, 4^th^ = 1.10–1.50 mm, 5^th^ = 1.60–2.70 mm, 6^th^ = 2.80–3.40 mm. In these experiments, all larval head capsule widths fell within these ranges. Weight of 1^st^ instar larvae was assumed to be 0.1 mg, based on a laboratory experiment taking the average weight of 10 neonate (< 24hr) *H*. *zea* larvae obtained from Benzon Research (Carlisle, PA). Larvae were weighed using a Mettler Toledo ML54T analytical balance.

Feeding location was measured both as categorical location (silk or tip+kernel in 2016; silk, tip, or kernel in 2017) and as distance penetrance into the ear (2017 only). Location was recorded based on where larvae were found to be actively feeding on a specific tissue. The ear penetrance rating used in this study was adapted from Wiseman and McMillian [33] and has been used in a number of papers to track larval feeding behavior in corn ears [34, 35]: 1 = larva feeding in upper half of silk channel, 2 = lower half of silk channel, 3 = silk around ear tip, 4+ = feeding distance (cm) down tip or kernels. Thus, a larva collected feeding in the lower half of the silk channel was given a rating of 2. A larva collected feeding 5 cm down from the tip of the ear was given a rating of 9 (4 + 5cm). This rating system does not account for lateral feeding movement or the linearity or compactness of feeding.

### Tissue sampling and Cry1F, Cry2A, and total protein quantification

Silk, ear tip, kernels, and husk tissue were collected from each plot in both years. Within each plot, five subsamples of each tissue were collected from separate ears and pooled. Silk tissue was collected twice each season. Early silk was collected when silks were freshly emerged from husk (<5 cm exposed) and still green. Late silk was collected 2–3 weeks later when ears were at the late milk stage (R3), after silks were pollinated, brown, and wilting. For early silk, late silk, and husk tissue, scissors were used to cut away the silk outside of the husk and then ~2.5 cm of husk, and the silk tissue within the husk, were cut from the ear and placed into plastic bags, and immediately frozen on dry ice. Scissors were sterilized and wiped clean with 70% ethanol in between each cutting. Silk within the husk, notably with the late-collected silk, were still moist and occasionally being fed on by larvae, as opposed to the dried down silk outside of the husk. The husk on the ear was then pulled back to expose the tip and kernels, and the top ~3 cm of the ear (tip tissue) was cut and placed in plastic bags and immediately frozen on dry ice. The ear tip tissue comprised only the unfertilized maternal tissue on the top ~3 cm of the ear, being careful not to include any fertilized kernels. The husk tissue used in further analyses was that harvested at the same time as the early silk tissue (R1) and only included the layers of husk tissue immediately surrounding the silk channel, and not the husk tissue on the exterior of the ear. Kernel tissue was harvested from R4 (dough) ears shortly after being transported from the field to the laboratory for injury rating. After kernels were harvested from ears of each plot, they were immediately frozen in a -20°C freezer until the kernels from remaining plots were harvested (~1 hr). After kernel sampling was complete, all samples were transferred to a -80°C freezer.

All tissue samples were stored in a -80°C freezer until being lyophilized, after which they were stored at -20°C with packets of silica gel desiccant (L2K Commerce Dry & Dry, Brea, CA). The five subsamples outlined above from each plot were pooled prior to grinding. Fifteen kernels (three kernels from each ear) were ground from each plot. For silk, tip, and husk tissue, tissue of approximately equal weight from each of five ears (subsamples) were pooled for each replicate. Lyophilized tissues were ground in 15 ml polycarbonate grinding vials with two 1.1 cm diameter stainless steel grinding balls (OPS Diagnostics, Lebanon, NJ) using a GenoGrinder 2010 (SPEX SamplePrep, Metuchen, NJ). Tissues were ground at 1500 RPM for the minimum time required to produce a fine powder (two minutes for silk, tip, and kernel tissue; seven minutes for husk tissue). After each tissue was ground into a powder, approximately 20 mg of tissue was transferred to a 1.5 ml microcentrifuge tube, with the weight recorded to the nearest 0.01 mg using a Mettler Toledo ML54T analytical balance. Protein extractions were performed based on the manufacturer’s instructions for each respective ELISA kit (see below). Proteins were extracted by adding 1000 ul 1X PBS-Tween 20 to ground tissue samples, vortexed for ~20 seconds, and then incubated at room temperature on a nutating mixer for 3 min. Tubes were centrifuged at 2152 rcf for 3 minutes and supernatant containing proteins was transferred to new, separate tubes for either enzyme-linked immunosorbent assay (ELISA) or Bradford assay, and immediately frozen at -80°C until each assay.

Prior to each protein assay, the supernatant was thawed and dilutions were made as necessary (as determined from practice trials). The concentration of Cry1F in silk, tip, kernel, and husk tissue was quantified for each Bt hybrid in the Pioneer family (Table 1) using a quantitative Cry1F ELISA kit (PSP 11700, Agdia, Inc., Elkhart, IN). The concentration of Cry2Ab2 in silk, tip, kernel, and husk tissue was quantified for each Bt hybrid in the Dekalb 2 family (Table 1) using a quantitative Cry2A ELISA kit (AP 005, Envirologix Inc., Portland, ME). Quantitative ELISA kits from these manufacturers have been used extensively in the literature to quantify several different Bt proteins in various plant, insect, and soil samples [36–41]. The concentration of total protein in silk, tip, kernel, and husk tissue was quantified for each Bt and non-Bt hybrid in the Pioneer and Dekalb 2 families (Table 1) using a standard Bradford assay protocol with bovine serum albumin (BSA) as the protein standard [42]. A Synergy H1 hybrid multi-mode microplate reader (BioTek Instruments, Inc., Winooski, VT) was used to measure the absorbance of Cry2A protein samples at 450 nm, Cry1F at 650 nm, and total protein samples at 595 nm. All samples were run in triplicate and only data points that fell within the standard curve were used in the analyses. Coefficients of variance between triplicate samples within plates never exceeded 10%.

### Statistical analysis

Linear mixed repeated measures models (PROC MIXED, [43]) were constructed for each hybrid family and year with proportion silks injured, number of silks injured, area of tip injured, area of kernels injured, number of ears injured, number of larvae per ear, and larval head capsule width as the dependent variables and hybrid, week, and their interaction as fixed effects. Proportion of silks injured and number of silks injured were highly correlated (PROC CORR; r = 0.94, df = 221, *P* < 0.0001), and only proportion of silks was used in further analyses. Replication was used as a random effect. Variance-covariance structure was specified as either compound symmetry, autoregressive, Toeplitz, unstructured, or ante-dependence, and selected based on lowest AICc value [44]. Models for number of larvae and head capsule width used autoregressive and compound symmetry variance-covariance structures, respectively. Residual plots were visually inspected for deviations in normality and constant variance. Proportion of silk injured was arcsine(sqrt(x)) transformed and number of ears injured, number of larvae, and head capsule width were log_10_(x+1) transformed before analysis to meet model assumptions. The denominator degrees of freedom were calculated following the methods of Kenward and Roger [45]. Mean separations were analyzed using Tukey’s honestly significant differences test [46] when significant at α < 0.05. The SLICE function was used to identify differences among hybrids within each sampling week when interactions were significant. Mean values for the 10 subsamples within each plot were calculated prior to statistical analyses, and data from some sampling periods were excluded from analyses because too few larvae were collected.

To analyze the feeding behavior of larvae in corn ears, two-way analysis of variance (PROC MIXED, [43]) was used as described below. Prior to ANOVA, data for number of larvae, head capsule width, and larval penetrance were averaged for the 10 subsamples collected within each replicate. For number of larvae and head capsule width, corn hybrid, tissue location (silk, tip, kernel), and their interaction were fixed effects, and replication was used as a random effect. Number of larvae was log_10_(x+1) transformed before analysis to meet model assumptions. For larval penetrance (2017 only), hybrid, instar, and their interaction were fixed effects and replication was used as a random effect; because of low numbers of larvae, instars were analyzed in three groups: 1^st^+2^nd^, 3^rd^+4^th^, and 5^th^+6^th^ instar.

For concentrations of Cry1F, Cry2A, and total protein in 2016 and 2017, tissue, year, and their interaction were fixed effects in a two-way analysis of variance with replicate used as a random effect (PROC MIXED, [43]). Cry1F was quantified for the three Pioneer hybrids expressing Cry1F, Cry1F + Cry1Ab, Cry1F + Cry1Ab + Vip3A. Cry2A was quantified for the two Dekalb 2 hybrids expressing Cry1A.105 + Cry2Ab2 and Cry1A.105 + Cry2Ab2 + Cry1F. Mean concentrations between hybrids each year were not significantly different and were pooled prior to analysis. Replication was used as a random effect. Mean separations were analyzed using Tukey’s honestly significant differences test [46] when significant at α<0.05.

## Results

In 2016, across nine Bt and non-Bt corn hybrids and seven weeks of sampling, 2,520 ears were sampled and 535 *H*. *zea* larvae were collected. In 2017, across seven corn hybrids and six weeks of sampling, 1,680 ears were sampled and 275 *H*. *zea* larvae were collected. In 2016, when larval instar could be determined, a total of 11, 70, 124, 115, 130, and 58 larvae for 1^st^-6^th^ instar, respectively, were collected. In 2017, when sampling began earlier in corn development but *H*. *zea* infestation was lower, 64, 50, 50, 40, 41, and 31 larvae for 1^st^-6^th^ instar, respectively, were collected.

### Feeding injury to silk, tip, and kernel tissues

For hybrid family Dekalb 1 (included in 2016 trials only), feeding injury in silk, tip, and kernels was not significantly different between the Bt and non-Bt hybrid (Table 2), although mean injury values for Cry1Ab corn were less than in the non-Bt hybrid for all three tissues most weeks. Maximum weekly feeding injury for Cry1Ab corn in silk, tip, and kernels was 34.0 ± 4.5% (mean ± SEM), 1.3 ± 0.3 cm^2^, and 1.6 ± 0.4 cm^2^, respectively. Maximum weekly feeding injury in the non-Bt hybrid in silk, tip, and kernels was 43.8 ± 6.4%, 2.6 ± 0.7 cm^2^, and 3.5 ± 0.3 cm^2^, respectively. As sampling progressed over time, injury to tip and kernel tissue, but not silk tissue, significantly increased.

**Table 2:** ANOVA statistics for *H*. *zea* Bt corn ear feeding in trials in Florence, South Carolina, 2016–2017.

Family^a^	Year	Factor	Proportion Silk Injured		Tip Area Injured (cm^2^)		Kernel Area Injured (cm^2^)	
			df	F^b^	df	F^b^	df	F^b^
Dekalb 1	2016	Hybrid	1, 5.72	1.67	1, 6	2.5	1, 6	5.62
		Week	3, 14.5	1.12	6, 36	5.5***	6, 36	5.29***
		Hybrid x Week	3, 14.5	0.33	6, 36	1.04	6, 36	1.08
Dekalb 2	2016	Hybrid	2, 17.5	39.5***	2, 9	132.27***	2, 34	132.71***
		Week	3, 20.7	8.05**	6, 54	4.08**	6, 48.4	10.95***
		Hybrid x Week	6, 21.2	1.11	12, 54	1.63	12, 49.6	9.63***
	2017	Hybrid	2, 6	22.52**	2, 14.1	36.91***	2, 11.5	4.3*
		Week	5, 45	15.11***	5, 12	21.24***	5, 10.3	7.1**
		Hybrid x Week	10, 45	1.21	10, 14.5	5.83**	10, 12.3	1.47
Pioneer	2016	Hybrid	3, 9.13	23.21***	3, 18.1	50.49***	3, 15	51.28***
		Week	6, 39.3	32.02***	6, 17	96.59***	6, 16.3	45.82***
		Hybrid x Week	9, 39.6	11.69***	18, 24	12.98***	18, 22.7	6.62***
	2017	Hybrid	3, 25.6	71.52***	3, 13.3	22.39***	3, 12	59.89***
		Week	5, 34.8	102.33***	5, 15.5	37***	5, 60	17.48***
		Hybrid x Week	15, 39.5	13.11***	15, 21.2	5.41***	15, 60	2.51**

^a^Family indicates grouping of near isolines of Bt and non-Bt hybrids.

^b^Asterisk indicates significance at *P* < 0.05 (*), *P* < 0.01 (**), and *P* < 0.001 (***)

**Fig 1:**
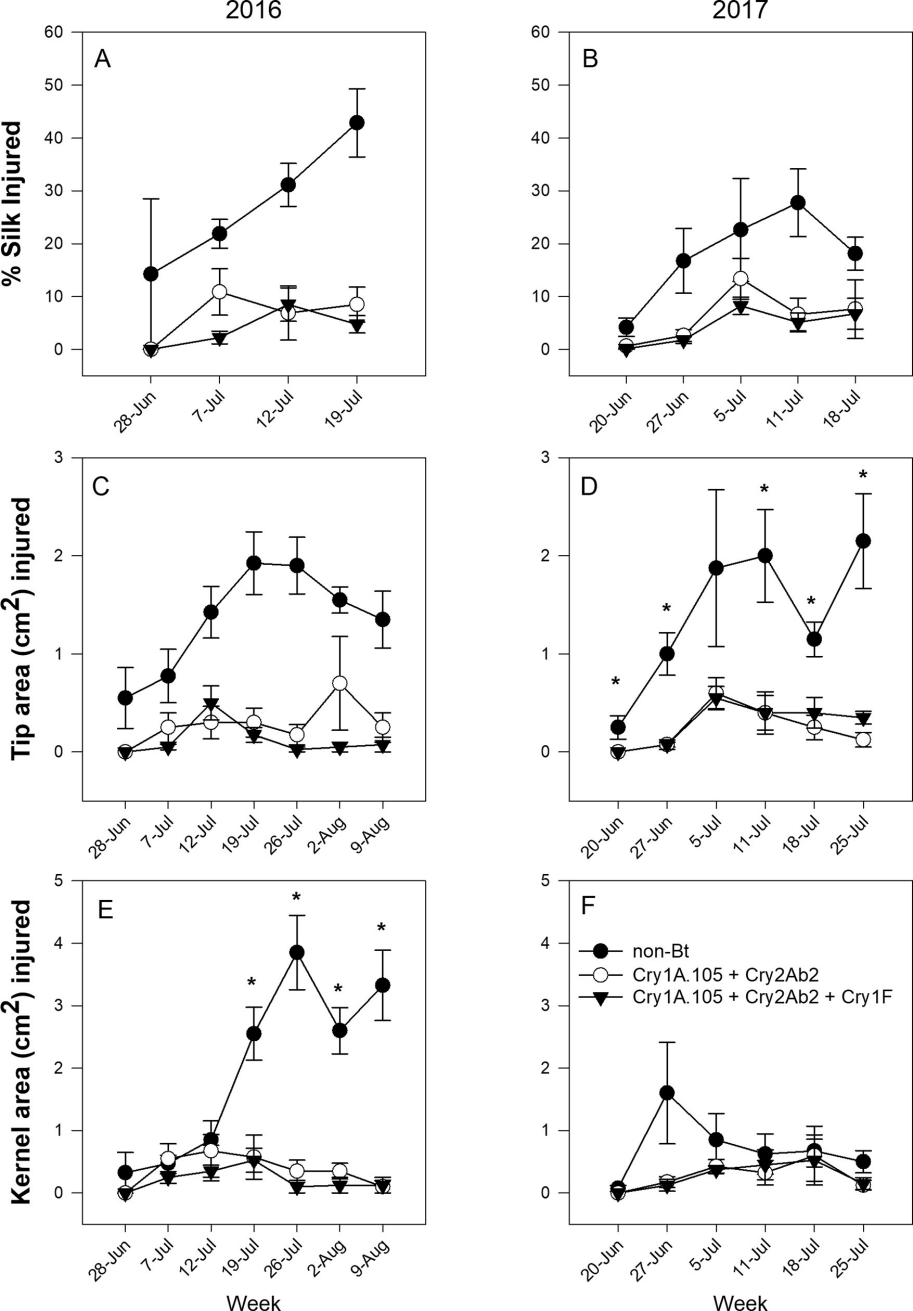
Mean *H*. *zea* feeding injury to silk (A, B), tip (C, D), and kernel (E, F) tissues in the Dekalb 2 hybrid family in Florence, South Carolina, in 2016 and 2017. Asterisk (*) indicates where SLICE function was used to identify significant differences among hybrids within each sampling week when the hybrid x week interaction was significant.

For hybrid family Dekalb 2 in both 2016 and 2017, feeding injury to silk, tip, and kernels was significantly reduced in both Bt hybrids (Table 2, Fig 1). Injury in ear tissues generally increased in the non-Bt hybrid as the season progressed but remained low for the Bt hybrids expressing Cry1A.105 + Cry2Ab2 and Cry1A.105 + Cry2Ab2 + Cry1F (Fig 1). Feeding injury among hybrids significantly interacted with sampling week for kernel and tip tissue in 2016 and 2017, respectively. Based on the SLICE function of PROC MIXED, differences in 2016 kernel injury only occurred beginning 19 July (Fig 1E). Tip injury in 2017 was significantly different all sampling weeks except on 5 July, when variability was highest in the non-Bt hybrid (Fig 1F).

**Fig 2:**
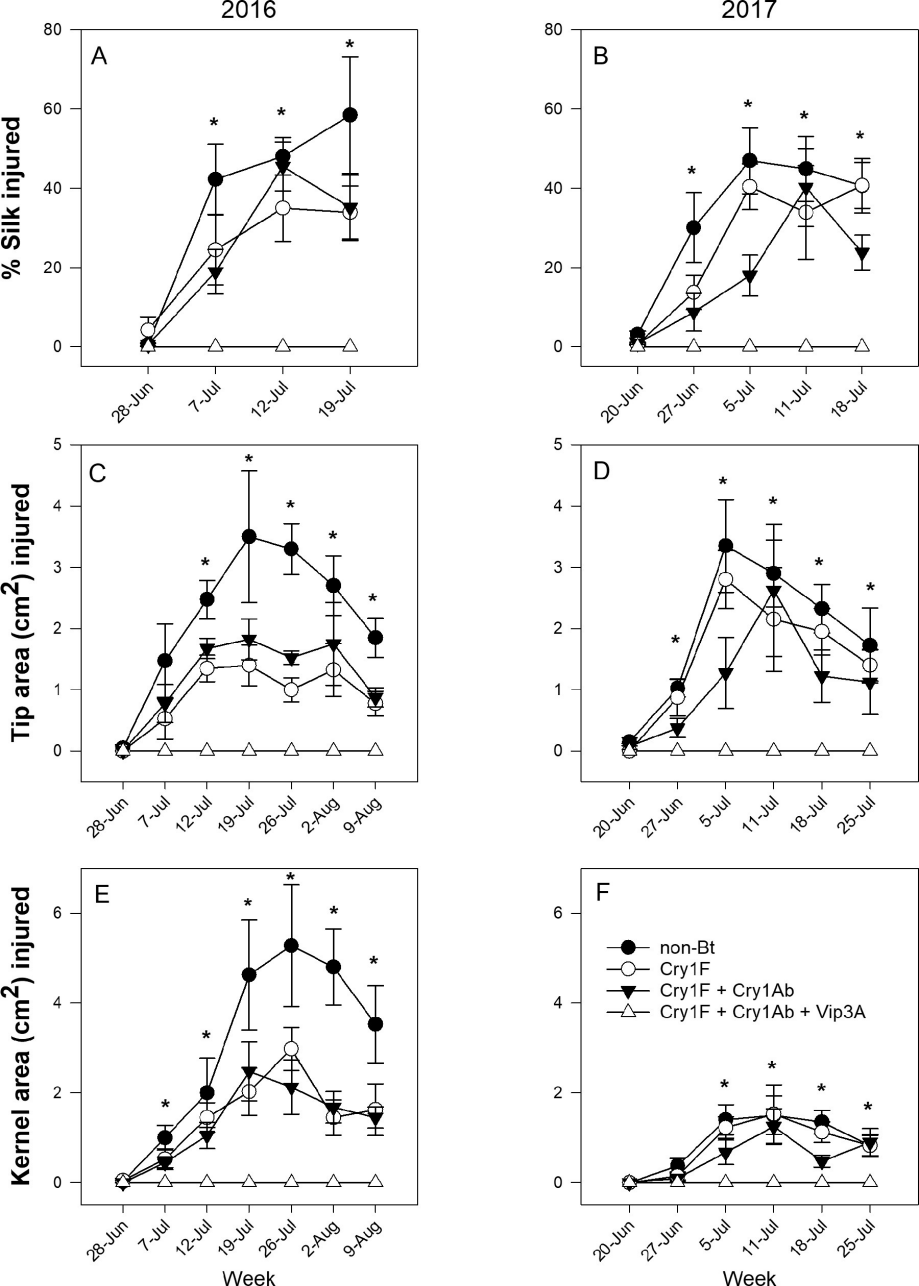
Mean *H*. *zea* feeding injury to silk (A, B), tip (C, D), and kernel (E, F) tissues in the Pioneer hybrid family in Florence, South Carolina, in 2016 and 2017. Asterisk (*) indicates where SLICE function was used to identify significant differences among hybrids within each sampling week when the hybrid x week interaction was significant.

For hybrid family Pioneer in both 2016 and 2017, Bt toxins in all three tissues significantly reduced feeding injury relative to tissues in the non-Bt hybrid (Table 2, Fig 2). This hybrid effect was driven largely by the hybrid producing the Vip3A toxin. In this hybrid, 520 ears were sampled across two years and only eight larvae were collected, with all larvae found in the silk, and silk injury never exceeding five individual silk strands per ear. There was no reported tip (Fig 2A, Fig 2B) or kernel injury (Fig 2E, Fig 2F) in the hybrid expressing Vip3A. Injury in all tissues both years significantly increased with time, but this effect interacted with hybrid due to injury differences not occurring until after the second sampling week. Based on the SLICE function of PROC MIXED, significant differences in tissue injury did not occur until the second or third sampling week (Fig 2).

The proportion of ears with feeding injury varied significantly among hybrids for all years and hybrid families except Dekalb 1 in 2016 (Table 3). In this trial, ear infestation peaked on 26 July with 83% of non-Bt ears and 60% of Cry1Ab ears with feeding injury to silk, tip, or kernels. In Dekalb 2 in 2016 during the week with the greatest number of ears injured (26 July), the hybrids producing Cry1A.105 + Cry2Ab2 and Cry1A.105 + Cry2Ab2 + Cry1F decreased the proportion of injured ears by 85 and 97%, respectively, compared with the non-Bt near isoline. In 2016 during the week with the greatest number of ears injured (19 July), the Pioneer hybrids producing Cry1F and Cry1F + Cry1Ab decreased the proportion of injured ears by 24 and 16%, respectively. Trends were similar in 2017, but to a lesser degree, due to reduced ear pressure from *H*. *zea*. In 2017, the highest recorded percent of injured ears was 68% of ears in the Pioneer non-Bt hybrid on 11 July. The proportion of ears injured significantly increased over the sampling period only in 2017.

**Table 3:** ANOVA statistics for *H*. *zea* characteristics in Bt corn trials in Florence, South Carolina, 2016–2017.

Family^a^	Year	Factor	Proportion Ears Injured		Larvae per 10 Ears		Head Capsule Width	
			df	F^b^	df	F^b^	df	F^b^
Dekalb 1	2016	Hybrid	1, 6	1.14	1, 15.6	1.37	1, 6.39	0.00
		Week	6, 36	2.23	6, 32.7	8.89***	4, 15.3	3.17*
		Hybrid x Week	6, 36	0.93	6, 32.7	1.27	4, 15.3	2.01
Dekalb 2	2016	Hybrid	2, 9	103.95***	2, 21.8	19.46***	2, 4.32	3.95
		Week	6, 14	3.48	6, 51.1	7.33***	3, 15.3	5.73*
		Hybrid x Week	12, 4.47	4.84	12, 51.4	3.44***	6, 15	3.94*
	2017	Hybrid	2, 8.79	16.11**	2, 17.2	0.61	2, 5.45	19.75*
		Week	5, 5	1.01	5, 40.4	17.94***	2, 14.3	46.38*
		Hybrid x Week	10, 5.72	1.21	10, 41.3	0.67	4, 14.2	1.80
Pioneer	2016	Hybrid	3, 25.8	326.58***	3, 22.9	29.33***	2, 10.9	5.33*
		Week	6, 17.9	12.51***	6, 69.6	34.3***	4, 30.7	32.33*
		Hybrid x Week	18, 25.1	1.95	18, 69.1	5.22***	8, 30.5	3.07*
	2017	Hybrid	3, 14.2	50.7***	3, 19.9	18.11***	2, 2.87	0.13
		Week	5, 51.6	2.69*	5, 52	17.61***	4, 24.4	28.20*
		Hybrid x Week	15, 52.9	1.13	15, 53.9	1.8	8, 25.3	2.96*

^a^Family indicates grouping of near isolines of Bt and non-Bt hybrids.

^b^Asterisk indicates significance at *P* < 0.05 (*), *P* < 0.01 (**), and *P* < 0.001 (***)

## Number of larvae per ear

**Fig 3:**
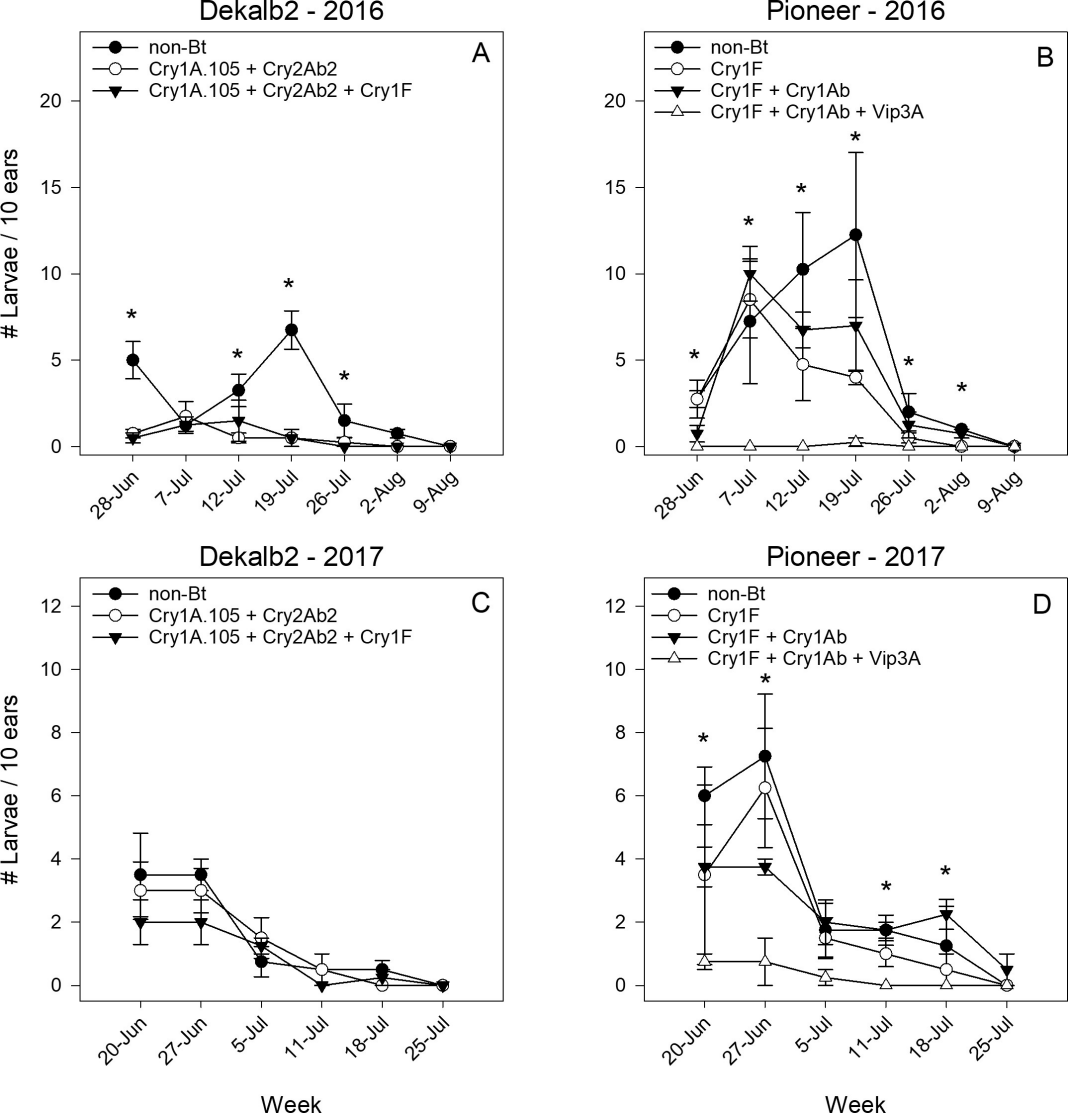
**Mean number of *H*. *zea* larvae per 10 ears for hybrids in the Dekalb 2 (A, C) and Pioneer (B, D) hybrid families in Florence, South Carolina, in 2016 and 2017.** Asterisk (*) indicates where SLICE function was used to identify significant differences among hybrids within each sampling week.

The number of larvae infesting ears was significantly reduced by the Bt hybrids in Dekalb 2 in 2016 and Pioneer in both years (Table 3, Fig 3). The number of larvae significantly decreased over time for each hybrid family during both years (Fig 3); however, there was a significant hybrid x week interaction for Dekalb 2 (Fig 3A) and Pioneer (Fig 3B) in 2016 due to a peak in larvae in the non-Bt hybrids on 19 July that did not occur in Bt hybrids. In family Dekalb 2, four of the first five sampling weeks in 2016 had significant variation in number of larvae among hybrids based on the SLICE function of PROC MIXED (Fig 3A), but this did not occur for a single week in 2017 (Fig 3C). In Pioneer, due to high mortality in the hybrid containing Vip3A, number of larvae varied significantly among hybrids nearly every individual week in each year (Fig 3B, Fig 3D).

## Larval head capsule width

**Fig 4:**
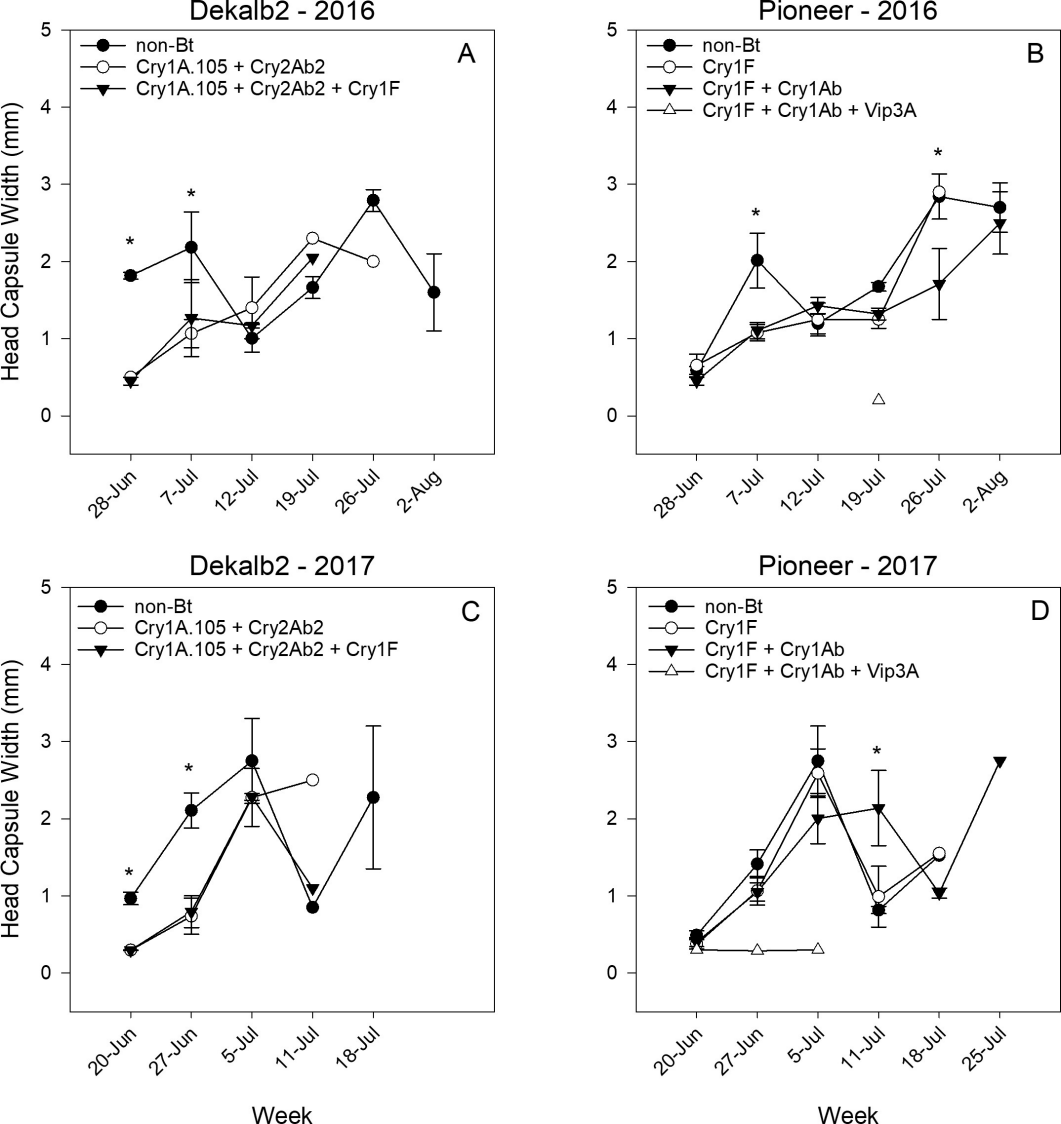
**Mean *H*. *zea* head capsule width for larvae collected in the Dekalb 2 (A, C) and Pioneer (B, D) hybrid families in Florence, South Carolina, in 2016 and 2017.** Asterisk (*) indicates where the SLICE function was used to identify significant differences among hybrids within each sampling week.

Mean head capsule width of larvae significantly increased over time for every hybrid and family in both years as larvae progressively developed to later instars (Table 3). There was a significant hybrid x week interaction in Dekalb 2 (Fig 4A) and Pioneer (Fig 4B) in 2016 due to significantly greater head capsule width in the non-Bt hybrid hybrids in the early sampling weeks. In Pioneer during 2017, the hybrid x week interaction reflected the significantly greater head capsule width in the hybrid Cry1F + Cry1Ab on 11 July, after the larvae in the non-Bt and Cry1F hybrid likely developed out of the ears (Fig 4D). In Dekalb 2 in 2017, mean head capsule width development in both Bt hybrids was approximately a week delayed relative to the non-Bt hybrid, as evident in the first three sampling dates (the only sampling dates statistically analyzed due to larval numbers) (Fig 4C).

## Tissue feeding location by larval age

**Fig 5:**
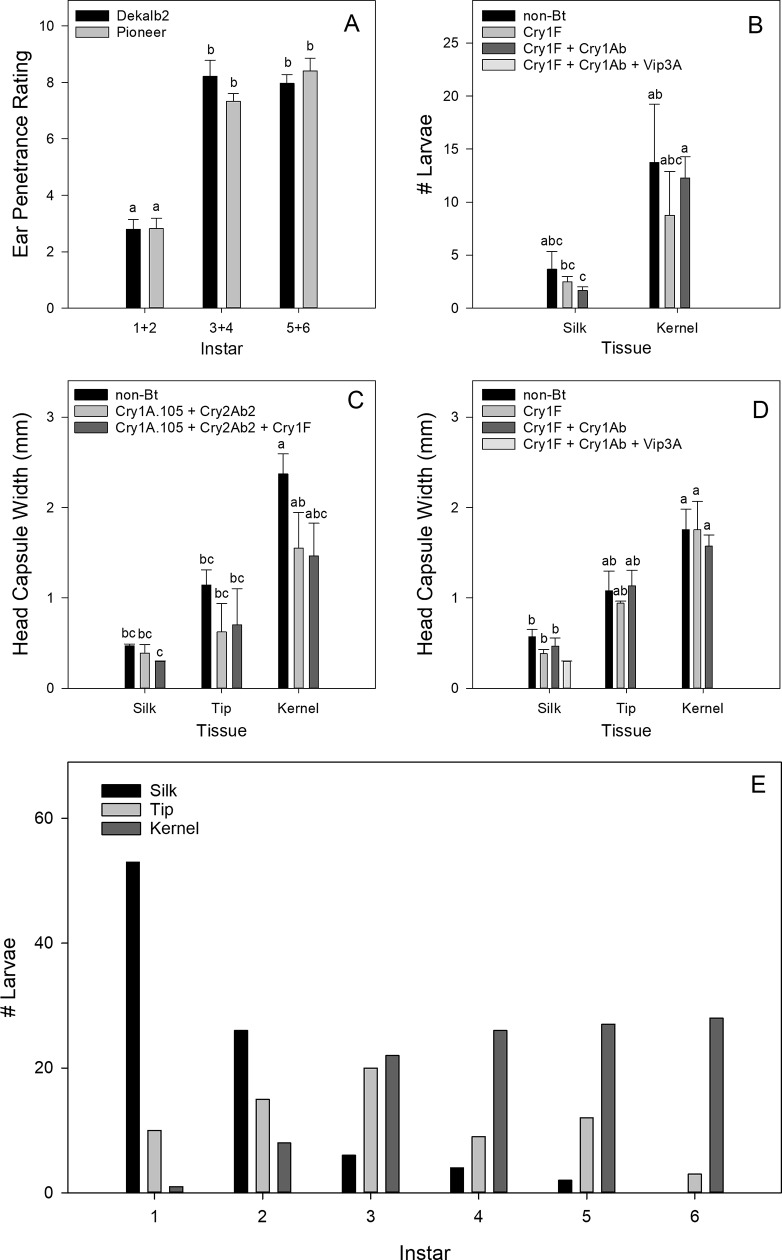
Age, number, and feeding location of *H*. *zea* larvae in ear tissues. (A) Distance larval feeding penetrance into corn ears of hybrids in the Dekalb 2 and Pioneer families by larval age group in 2017. (B) Mean number of larvae collected in either silk or kernel tissue in hybrid family Pioneer in 2016. (C) Mean larval head capsule width in silk, tip, and kernel tissue in hybrid family Dekalb 2 in 2017. (D) Mean larval head capsule width in silk, tip, and kernel tissue in hybrid family Pioneer in 2017. (E) Sum of larvae collected across all Bt and non-Bt corn hybrids in silk, tip, or kernel tissue by instar in 2017.

Larval feeding penetrance into the ear (included only in 2017) did not significantly differ among Bt and non-Bt hybrids for either Dekalb 2 (F = 0.13, df = 2, 18, *P* = 0.882) or Pioneer (F = 0.76, df = 2, 23, *P* = 0.479), but did differ among instar groups for both Dekalb 2 (F = 54.72, df = 2, 18, *P* < 0.0001) and Pioneer (F = 55.77, df = 2, 23, *P* < 0.0001). For both hybrid families (combining the Bt and non-Bt hybrids), instar groups 3^rd^+4^th^ and 5^th^+6^th^ fed significantly deeper into corn ears (mean penetrance rating ranging from 7.3 to 8.4) than instar group 1^st^+2^nd^ (mean penetrance rating ranging from 2.80 to 2.83) (Fig 5A). This rating equates to 1^st^+2^nd^ instars feeding as deep as silk around the ear tip, and later instars all feeding, on average, approximately 4 cm down from the tip of the ear.

The number of larvae feeding in either the silk or kernels (analyzed only for Pioneer in 2016) or the silk, tip, or kernels (2017) did not vary significantly among hybrid for any family either year (Table 4). Within each tissue location, the number of larvae in the Bt hybrids was generally less than in the non-Bt hybrid, but this difference was not significant (Fig 5B). Differences in number of larvae did significantly vary by tissue location (Pioneer, 2016), with more larvae collected on kernels than on silks (Table 4, Fig 5B). This might be a result of either initiating sampling later in the development of *H*. *zea* (after larvae had moved out of the silk into the ear), or as a result of sampling error (not detecting all the larvae feeding in the silk).

**Table 4:** ANOVA statistics for *H*. *zea* larvae characteristics by tissue location (Florence, SC, 2016–2017).

Family^a^	Year	Factor	N larvae			Head Capsule Width		
			df	F^b^	P	df	F^b^	P
Dekalb 2	2017	Hybrid	2, 19	1.89	0.1788	2, 19	3.30	0.0587
		Location	2, 19	2.27	0.1306	2, 19	24.55	**< .0001**
		Hybrid x Location	4, 19	0.87	0.5027	4, 19	0.67	0.6229
Pioneer	2016	Hybrid	2, 13	0.57	0.5800	2, 13	1.95	0.1811
		Location	1, 13	24.21	**0.0003**	1, 13	3.33	0.0910
		Hybrid x Location	2, 13	0.97	0.4065	2, 13	3.01	0.0843
	2017	Hybrid	2, 23	1.38	0.2722	2, 23	0.41	0.6676
		Location	2, 23	1.45	0.2552	2, 23	41.94	**< .0001**
		Hybrid x Location	4, 23	1.06	0.4005	4, 23	0.41	0.7993

^a^Family indicates grouping of near isolines of Bt and non-Bt hybrids.

^b^Dekalb 1 and Dekalb 2 2016 data omitted because of lack of data.

The head capsule width of larvae feeding in different tissues did not vary by hybrid for any hybrid family in 2016 and 2017, indicating that, regardless of Bt or non-Bt hybrid, larval age was the same in each tissue (Table 4). Head capsule width did significantly vary among tissue location in Dekalb 2 or Pioneer in 2017 (Fig 5C and 5D), and supported the penetrance data that larvae feeding in the tip and kernel are older than larvae feeding in the silk. Based on head capsule width for Dekalb 2 in 2017, the average instar feeding in the silk, tip, and kernels was 1^st^, 3^rd^, and 5^th^ instar, respectively. For Pioneer in 2017, the average instar feeding in the silk, tip, and kernels was 2^nd^, 3^rd^, and 5^th^ instar, respectively. However, when number of larvae of each instar in each tissue were summed across all 2017 trials, it illustrated that each tissue contained varying proportions of each instar (Fig 5E).

## Cry1F and Cry2A tissue concentrations using ELISA

**Fig 6:**
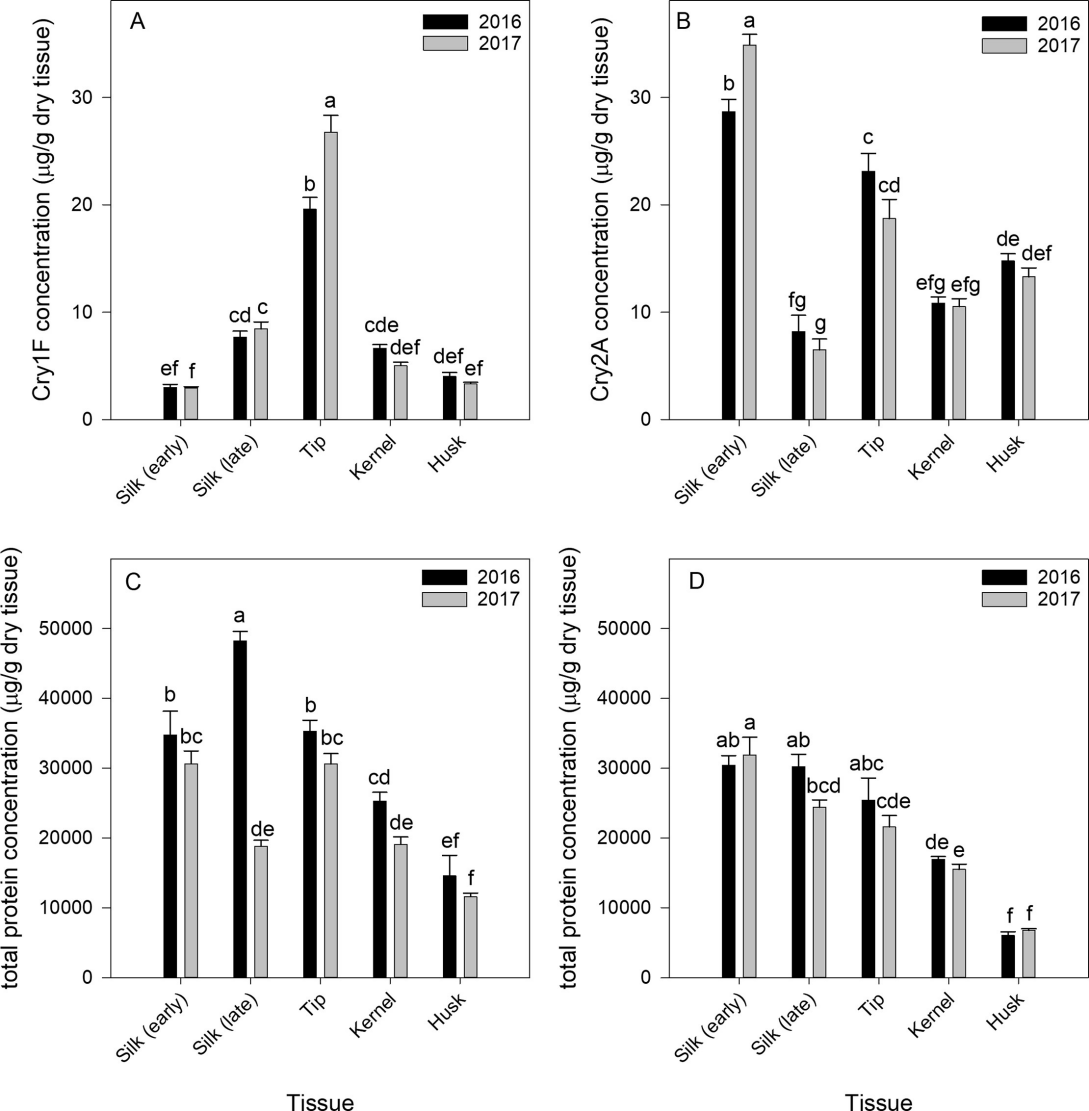
Mean concentration of Cry1F and total protein among tissue types for hybrid family Pioneer in Florence, South Carolina, in 2016 and 2017 (A, C). Mean concentration of Cry2A and total protein among tissue types for hybrid family Dekalb 2 in 2016 and 2017 (B, D). Individual Bt hybrids within each family and year (Table 1) were not significantly different and pooled.

The concentration of Cry1F in dry tissue from the three Pioneer hybrids varied significantly among tissues (F = 238.47, df = 4, 95, *P* < 0.0001) and year (F = 4.97, df = 1, 95, *P* = 0.028). The tissue x year interaction was significant (F = 11.11, df = 4, 95, *P* < 0.0001) due to tissue concentrations between years varying in the tip but not in any other tissue. Tip tissue had the highest concentration of Cry1F in both years, but also the highest variability between years, as concentration in 2017 was 1.4-fold higher than the concentration in 2016 (Fig 6A). Larvae feeding in silk tissue would be exposed to 2.7-fold higher concentrations of Cry1F in older silk than freshly emerged silk. The concentration of total protein in dry tissues from these same Pioneer hybrids also varied significantly among tissues (F = 61.01, df = 4, 95, *P* < 0.0001), year (F = 91.29, df = 1, 95, *P* < 0.0001), and their interaction (F = 29.0, df = 4, 95, *P* < 0.0001). Total protein concentration varied the most in older silk tissue, where the concentration was highest overall in 2016 but much lower in 2017 (Fig 6C).

The concentration of Cry2A in dry tissue from the Dekalb 2 hybrids producing Cry1A.105 + Cry2Ab2 and Cry1A.105 + Cry2Ab2 + Cry1F varied significantly only among tissues (F = 161.18, df = 4, 61, P < 0.0001). The tissue x year interaction (F = 6.73, df = 4, 61, *P* = 0.0001) reflected the significant variation in Cry2A concentration in early silk between 2016 and 2017. The highest concentration of Cry2A was found in early silk tissue both years, followed by the tip tissue (Fig 6B). Older silks had the lowest concentration of Cry2A. The concentration of total protein in dry tissues from these same two hybrids varied significantly among tissues (F = 68.49, df = 4, 61, P < 0.0001) but not years (F = 1.38, df = 1, 6, *P* = 0.285) or their interaction (F = 1.82, df = 4, 61, *P* = 0.137). Total protein concentration was highest in the silk and tip tissue (Fig 6D).

## Discussion

Larvae of *H*. *zea* are exposed in ear tissues of Bt corn to a heterogeneous distribution of Bt toxins. Differences in toxin concentrations can influence the dominance of resistance and provide the opportunity for behavioral responses, such as aversion, which can influence the evolution of resistance to these toxins [19, 21, 25–28, 47]. Previous investigations into the behavior of *H*. *zea* feeding on ears in field experiments of Bt corn have been limited to recording injury to kernels [30, 31], despite the fact that larvae feed in all major ear tissues—silk, tip, and kernels—and that concentration of Bt toxins varies by tissue type, tissue age, and environmental condition [20, 21]. The main objective of these experiments was to test the null hypothesis that age- and tissue-specific feeding behavior of *H*. *zea* is the same on Bt and non-Bt hybrids. Our study provides a detailed account of where and when larvae are feeding in corn ears of different Bt and non-Bt hybrids, in addition to quantifying the concentration of Cry2Ab2 and Cry1F in those same ear tissues. We found no significant difference in age-specific feeding location between Bt and non-Bt hybrids and fail to reject our null hypothesis. This could be due to low toxicity in *H*. *zea* to some of the Bt toxins expressed in the hybrids used this study, to development of resistance in *H*. *zea* to these toxins, or a combination of both.

We measured the instar of all larvae collected in each ear tissue location: silk, tip, and kernels (in 2017 only). The head capsule width (an indicator of instar or age) for larvae in each tissue did not significantly differ between Bt and non-Bt hybrids. In the silk, tip, and kernels, the mean instar was 1^st^, 3^rd^, 5^th^ and 2^nd^, 3^rd^, 5^th^, averaged across hybrids in the Dekalb 2 and Pioneer families, respectively. When head capsule width was analyzed over time, larval development was significantly delayed in the Bt hybrids in Pioneer 2016 and Dekalb 2 2017 (Table 3, Fig 4B, Fig 4C). In later weeks, mean head capsule width often decreased, indicating either older larvae finished development and cycled out of corn, a second generation of corn earworm oviposited and was developing, or a combination of both. In Dekalb 2 2017, larval age in the hybrids producing Cry1A.105 + Cry2Ab2 and Cry1A.105 + Cry2Ab2 + Cry1F was delayed by approximately one week compared with development on a non-Bt near isoline. Considered alongside our age-location data, this shows that developmentally delayed larvae on these Bt hybrids remain feeding longer in the same tissues common to that age group compared to a non-Bt near isoline. When we analyzed the distance of individual larval penetrance into the ear, our age-location results were corroborated: age-specific penetrance did not differ among hybrids, with 1^st^+2^nd^ instars in the silk and both 3^rd^+4^th^ and 5^th^+6^th^ instars feeding approximately 4 cm down from the tip of the ear. The penetrance data do not account for the fact that the size of the tip of the ear (defined here as unfertilized, F_1_ maternal tissue) is not constant among all ears. The tip usually comprises the first ~3 cm of the ear distal to the ear shank, but some ears develop fertilized kernels all the way to the top of the ear with very little tip area exposed. Likewise, ears can suffer poor pollination due to heat stress, drought, and insect feeding [48, 49], and this can result in significant reductions in kernel fertilization and yield loss. It is unknown what effect poor pollination has on larval feeding penetrance in the ear. In one of the few other studies to investigate age-specific feeding location, Archer and Bynum [17] concluded that kernel feeding begins when *H*. *zea* larvae reach 3^rd^ instar. Their study did not differentiate tip tissue from kernel tissue and so our results—that 3^rd^ instars are the most frequently found instars on tip tissue—likely support their findings.

Despite failing to reject our null hypothesis that age- and tissue-specific feeding behavior of *H*. *zea* is the same on Bt and non-Bt hybrids, it is worth noting the trend for smaller mean head capsule width in each tissue for nearly every Bt hybrid compared with the non-Bt control (Fig 5C and 5D), while the number of larvae collected in each tissue was the same (Table 4). This would indicate that the larvae feeding in each Bt hybrid tissue may be younger than conspecifics feeding in the same non-Bt tissues, either because larvae of each instar are smaller or that younger larvae are moving into tip and kernel tissues earlier in Bt compared with non-Bt ears. Additional experiments are required to know if such an effect exists. In Horner et al. [31], it was demonstrated that the spatial patterns of damaged kernels in Bt (Cry1Ab) corn ears by larvae (instars 3^rd^-6^th^) were “significantly less compact, more linear, and had a greater perimeter length.” The only data the authors presented relating to ear penetrance were that Bt ears had less frequent feeding damage extending into the upper ear below the tip, compared with that observed on non-Bt ears. However, these data were not statistically analyzed. Dowd [30] reported that the feeding behavior of “railroading” (small larvae partially damaging many kernels along a silk channel) occurred at varying rates in Bt hybrids expressing high levels of Cry1Ab, but not in non-Bt (or low expressing event) hybrids.

In addition to our analyses of feeding penetrance and larval age by location, we summed all larvae of each instar in each tissue location (Fig 5E) to demonstrate that varying proportions of each instar are present in each tissue. These data, although not statistically analyzed and combined across all Bt and non-Bt hybrids, reveal that 2^nd^ instars feed in all three tissues, 3^rd^ instars feed almost equally between tip and kernels, and that 4^th^ and 5^th^ instars do most of their feeding in both the tip and kernels. The reason age-specific feeding location matters in Bt ears (or non-Bt ears pollinated with Bt pollen) is that susceptibility to Bt toxins can vary with instar. Differences in susceptibility among larval instars to Bt toxins have been reported by a number of authors. Studies with *H*. *zea* have found that younger instars are more susceptible than older instars [50], but in studies with related species the interaction between age and susceptibility can differ and depends on the insect species and the Bt protein used [51, 52]. Larval mortality from Bt toxins can also be reduced if larvae are able to move from Bt tissue to non-Bt tissue. Dulmage et al. [53] fed *Chloridea virescens* (Fabricius), a species closely related to *H*. *zea*, Bt-treated diet *ad libitum* for one, two, or three days before moving larvae to a non-Bt diet and remarked on the “unexpected capacity to recover from the effects of [Bt].” A detailed study of *H*. *armigera* feeding behavior in choice bioassays of Bt and non-Bt treated artificial diet demonstrated larvae could not detect Bt and only avoided it post ingestion [54]. Recovery and aversion post ingestion of Bt is less likely as protein toxicity increases, such as with toxins that approach high dose. In our study, only eight larvae were collected from 520 ears of the hybrid producing the Vip3A toxin, and not a single larva had fed on more than five individual silk strands before dying. However, as populations develop resistance to Bt toxins (and if *H*. *zea* larvae truly are capable of behavioral responses such as avoidance or aversion), larvae will be able to tolerate higher doses and have more opportunities to move into non-Bt tissue or tissue with less Bt concentrations.

In addition to instar, susceptibility to Bt toxins can also be influenced by environmental factors, such as dietary nutrition. Deans et al. [55] demonstrated that both the protein: carbohydrate (P:C) ratio and total macronutrient concentration of a diet significantly interacted with *H*. *zea* susceptibility to Cry1Ac. *H*. *zea* self-selects and develops best on protein-biased diets [56], but when fed on a Cry1Ac-treated, carbohydrate-biased diet, there was a 100-fold decrease in susceptibility. Our study details the variation in Cry protein and total protein concentrations among tissue type, and Deans et al. [57] showed that both total macronutrient content and P:C varied among silk, husk, and kernel type. In Bt or Bt-pollinated refuge corn ears, larvae of *H*. *zea* will differentially encounter Cry toxin concentrations relative to tissues with varying nutritional qualities and this may influence both susceptibility and feeding behavior. Larval feeding behavior is likely influenced by a combination of aversion to Bt and nutritional regulation [58]. *H*. *zea* larval nutrition, development, and survival can also be influenced by cannibalism [59], which in turn may be influenced by exposure to Bt when larvae are not resistant [60, 61]. While cannibalism can be an important nutritional factor for developing *H*. *zea* larvae, it likely had less influence in this study compared to others because of lower ear infestations and densities [62, 63]. Future experiments should include more detailed studies of how *H*. *zea* regulates nutrition among ear tissue type with and without Cry proteins, and how this effect is further influenced by larval age and larval densities (i.e. cannibalism).

*H*. *zea* is a target pest for both Cry2Ab2 (+ Cry1A.105) and Cry1F corn [21, 64]. Cry2Ab2 is significantly more toxic to *H*. zea than Cry1F, which often does not reduce survival of *H*. *zea* or infestation in the field [16, 65], although larvae collected feeding on Cry1F corn ears can have significant reductions in pupal weight compared with those that fed on non-Bt corn [63, 66]. As hybrids in our study expressed either single (Cry1F or Cry1Ab) or multiple toxins (combinations of (Cry1F, Cry1A.105, Cry2Ab2, and Vip3Aa20), *H*. *zea* was affected in some hybrids by Bt toxins that we did not quantify, as we had limitations on commercially available ELISA kits for quantifying Bt toxins in plant tissues rather than just detection. However, our data provide evidence of significant variability among tissues in two Bt toxins that are known to significantly affect the survival and biology of *H*. *zea* [63, 66]. Our goal was to determine the relative concentrations of Cry1F and Cry2Ab2 among tissues and at different times. Concentrations of Cry1F and Cry2Ab2 in Pioneer and Dekalb 2, respectively, varied significantly among tissue type. In the Pioneer hybrids, Cry1F concentration was highest in tip tissue during both years by more than two-fold compared with any other tissue. Late silk (harvested at R3) had significantly higher concentrations of Cry1F than early silks (R1). In Dekalb 2, the reverse was true, and early silk also had higher Cry2Ab2 concentrations than any other tissue. Our results cannot be directly compared to other studies due to differences in methodology (e.g. sampling tissue at a different stage) or data availability, but where the data are available they align with our results in relative tissue concentrations. Cry1F tissue-specific concentrations in corn showed that concentrations in silk (age not listed) were the lowest of all tissues tested (e.g. leaf, grain, stalk, pollen) [67]. While experiments quantifying Cry2A concentrations in space and time are more prevalent in cotton, ELISA data submitted to the EPA as part of the registration of Cry2Ab2 in corn showed concentrations in silk (R1) were higher than in most other tissues tested (e.g. forage, grain, pollen) except leaves [21]. To the best of our knowledge, our study is the first to quantify the concentrations of Cry1F and Cry2Ab2 toxins in silk tissue of Bt corn hybrids at two different time points, pre- and post-fertilization, capturing the variation in toxin exposure made possible to *H*. *zea* larvae by their duration of oviposition [17]. Additionally, we could find no other study quantifying the concentrations of these toxins in maternal tip tissue of Bt hybrids, despite it being a distinct and important feeding location for larvae. Our ELISA results demonstrate the heterogeneous nature of Bt protein production in the silk, tip, and kernels and reveal that the development and survival of larvae in these tissues is likely influenced by instar, although we did not detect behavioral differences resulting from exposure to Bt toxins in this study. Each instar (1^st^-6^th^) tends to be found feeding in one or more tissues with early and later instars more likely to be found feeding in the silk and kernels, respectively.

Our results regarding age- and tissue-specific feeding behavior fill important knowledge gaps needed for modeling the development of *H*. *zea* resistance on Bt ears and non-Bt ears fertilized by Bt pollen. Our data show that kernel feeding primarily begins with 3^rd^ instars, but it is not until the 4^th^ instar that the majority of larvae are found feeding on kernels. Additionally, a proportion of 3^rd^, 4^th^, and 5^th^ instars continue to feed, at least partially, on maternal tip tissue (Fig 5C, 5D and 5E). These instars are likely not equally susceptible to Bt toxins, and varying proportions of larvae within each instar will be exposed to different tissue-specific nutrition and Bt concentrations. Onstad et al. [68] proposed that the timing of larval movement relative to age should be considered in most studies of seed blends for IRM. One reason for this, as the authors state, is that if fertilized kernels are only fed on by older larvae then even susceptible larvae may not be significantly harmed by the dose of toxins in kernels. Our results provide these data and lay the groundwork to further study the impact of heterogeneous Bt expression on age-specific survival and development.

A seed mixture of Bt and non-Bt corn is one proposed strategy for managing resistance by insects to Bt corn rather than a separate block of non-Bt refuge corn. On Bt ears, the kernels can consist of a mosaic of Bt and non-Bt phenotypes [19, 22], which may influence larval exposure and survival. For ear-feeding pests on non-Bt refuge ears in seed mixtures, a major concern is cross-pollination by pollen from surrounding Bt plants. This concern was a focal point of the July 2018 report published by the FIFRA Scientific Advisory Panel regarding resistance of Lepidopteran pests to Bt plant incorporated protectants (PIPs) [69]. The final report stated that seed blends decrease the durability of PIPs to resistance evolution in ear-feeding lepidopteran pests compared to a structured refuge, and the panel recommended that seed blends not be used in the southern United States where *H*. *zea* overwinters. The report also noted that in order to understand resistance evolution of *H*. *zea* in seed mixtures, it is essential to determine the survival of heterozygotes (rS) on refuge plants expressing Bt in some kernels. Since this information is not available, it was noted that it is still informative to instead consider data on the survival of susceptible *H*. *zea* under different scenarios. From our data, we posit that one such scenario affecting larval survival in refuge ears is the age-distribution of larvae feeding in kernels. Our findings show that selection pressure from cross-pollination of refuge ears will primarily affect *H*. *zea* larvae 3^rd^ instar and older. Depending on the timing of oviposition, the rate of development, and natural variation in larval movement and feeding behavior, kernel feeding can be initiated at different instars, and these instars will be differentially susceptible to Bt toxins in kernels, which in turn will influence survival and/or fitness. Onstad et al. [68] summarized 14 field trials measuring survival of *H*. *zea* larvae on refuge ears in seed mixtures and found that relative survival (compared with larvae on ears of pure stands of non-Bt corn) varied over time and location, ranging from 0.37 to 1.0 (mean = 0.82). They hypothesized that the effects of non-Bt ears contaminated with Bt pollen are influenced by factors such as “weather, corn hybrid, cultivation practices, *H*. *zea* behavior, and synchrony of insect flights with a certain plant development stage.” These differences are likely due, in part, to differences in age-distribution of larvae in contaminated refuge ears. Because younger instars generally do not feed in kernels, they would typically be free from Bt selection in a blended refuge ear. While this provides some evidence in support of a blended refuge, it is still unknown at what age larval survival is no longer affected by exposure to Bt in refuge kernels. If, only after maturing to the 4^th^ instar while having fed on non-Bt silk and tip tissue, larval survival and fitness is no longer reduced by feeding on Bt kernels, then only the smaller proportion of 2^nd^ and 3^rd^ instars feeding on kernels will have reduced survival, delayed development, or another negative effect. Additional studies should determine if the age- and tissue-specific distribution we report (Fig 5C, 5D and 5E) is similar in other years, regions, and agricultural landscapes to fully understand the implications on effectiveness of blended refuge as an IRM strategy.

Additional objectives of our study were to compare feeding injury to silk, tip, and kernel tissues, infestation, and larval survival between Bt and non-Bt hybrids. To the best of our knowledge, this study is the first to measure and quantify silk feeding by *H*. *zea* in the field, and silk feeding plays a major role in the successful establishment of *H*. *zea* neonates. We measured both the number of silks injured and the proportion of silks injured, and both variables were highly correlated. Data on injury to silks generally mirrored that of tip and kernel injury (Table 2). Our experiments showed that Cry1Ab in the Dekalb 1 hybrid did not significantly reduce injury to any tissue, infestation, or larval survival (figure not shown). Earlier studies in the same geographic region investigating the effects of Cry1Ab on *H*. *zea* found that kernel injury was reduced, and prepupal survival was reduced 60–85%, with additional mortality occurring during the pupal stage [62]. More recent studies have reported no effect or decreasing efficacy of Cry1Ab on survival and kernel injury [13, 63]. In our study, it is possible that differences in survival could have been detected by monitoring larvae through pupation, as pupal effects, such as weight reduction, are still prevalent [66]. Concentrations of pyramided Bt proteins present in Dekalb 2 and Pioneer hybrids significantly reduced injury in all tissues and also reduced ear infestation and larval survival. The development of resistance to the Dekalb 2 Bt proteins Cry1A.105 and Cry2Ab2 has recently been reported [13, 16, 70], but the results reported here demonstrate these toxins still reduce feeding injury and larval survival. The effects of Cry1F and Cry1F + Cry1Ab in the Pioneer hybrids on ear injury, infestation, and survival were not as apparent relative to those of Cry1A.105 + Cry2Ab2, and hybrid effects in the Pioneer hybrids were driven primarily by the Vip3A pyramid. This hybrid had no tip or kernel injury, and no larvae survived past the 1^st^ instar, mirroring the results of other studies [63, 71, 72]. However, successful development of *H*. *zea* has recently been reported in Vip3A pyramids [66], in addition to high levels of H. zea injury to corn expressing Vip3A in a trial in Texas [73].

In conclusion, we characterized the age-specific feeding behavior of *H*. *zea* in Bt and non-Bt corn hybrids. We quantified the concentration of Cry1F and Cry2Ab2 to demonstrate the spatial and temporal heterogeneity of toxin distribution in silk, tip, and kernel tissues that can affect larval exposure to Bt toxins and create the opportunity for behavioral responses. Hybrids containing Vip3A were highly efficacious at reducing infestation, survival, tissue injury, and larval movement. However, *H*. *zea* resistance to Cry1A and Cry2A toxins has developed in the mid-Atlantic, southeastern, and mid-southern United States [13, 15, 16, 71], underlining the intense selection pressure placed on Vip3A, even in pyramids expressing Cry1A and Cry2A toxins. Future studies should quantify the spatial and temporal concentrations of Vip3A in ear tissues and determine how these concentrations vary with environmental factors. Future transgenic plants with next-generation insect control technologies should be investigated for their toxin heterogeneity and effects on larval movement in ears to accurately assess the risk of resistance. Finally, we did not detect significant changes in age-specific feeding behavior of *H*. *zea* between ear tissues, but, given the behavioral changes on kernels and artificial diets reported in other studies, feeding behavior of *H*. *zea* on corn ears should continue to be investigated with current and future transgenic insecticidal toxins to ensure all factors influencing the development of resistance to Bt toxins are fully realized and to understand under what circumstances IRM strategies, such as seed mixtures, will delay or hasten the evolution of resistance.

## References

[1] Fitt GP. The ecology of *Heliothis* species in relation to agroecosystems. Annu Rev Entomol 1989;34(1):17–53.

[2] US EPA. Current and previously registered section 3 Plant-Incorporated Protectant (PIP) registrations. 2018; Available at: https://www.epa.gov/ingredients-used-pesticide-products/current-and-previously-registered-section-3-plant-incorporated. Accessed 1/2, 2019.

[3] Cattaneo MG, Yafuso C, Schmidt C, Huang CY, Rahman M, Olson C, et al Farm-scale evaluation of the impacts of transgenic cotton on biodiversity, pesticide use, and yield. Proc Natl Acad Sci U S A 2006 5 16;103(20):7571–7576. 10.1073/pnas.0508312103 16675554 PMC1457091

[4] Marvier M, McCreedy C, Regetz J, Kareiva P. A meta-analysis of effects of Bt cotton and maize on nontarget invertebrates. Science 2007 6 8;316(5830):1475–1477. 10.1126/science.1139208 17556584

[5] Hutchison WD, Burkness EC, Mitchell PD, Moon RD, Leslie TW, Fleischer SJ, et al Areawide suppression of European corn borer with Bt maize reaps savings to non-Bt maize growers. Science 2010 10 8;330(6001):222–225. 10.1126/science.1190242 20929774

[6] US EPA. Biopesticides registration action document, Cry1Ab and Cry1F *Bacillus thuringiensis* (Bt) corn plant-incorporated protectants. 2010; Available at: https://www3.epa.gov/pesticides/chem_search/reg_actions/registration/decision_PC-006481_1-Sep-10.pdf. Accessed 1/2, 2019.

[7] Lu Y, Wu K, Jiang Y, Guo Y, Desneux N. Widespread adoption of Bt cotton and insecticide decrease promotes biocontrol services. Nature 2012;487(7407):362 10.1038/nature11153 22722864

[8] Perry ED, Ciliberto F, Hennessy DA, Moschini G. Genetically engineered crops and pesticide use in US maize and soybeans. Science advances 2016;2(8):e1600850 10.1126/sciadv.1600850 27652335 PMC5020710

[9] Dively GP, Venugopal PD, Bean D, Whalen J, Holmstrom K, Kuhar TP, et al Regional pest suppression associated with widespread Bt maize adoption benefits vegetable growers. Proc Natl Acad Sci U S A 2018 3 27;115(13):3320–3325. 10.1073/pnas.1720692115 29531049 PMC5879701

[10] U. S. Department of Agriculture-Economic Research Service. Adoption of genetically engineered crops in the U.S. 2018; Available at: https://www.ers.usda.gov/data-products/adoption-of-genetically-engineered-crops-in-the-us.asp. Accessed 1/3, 2019.

[11] Gould F. Sustainability of transgenic insecticidal cultivars: integrating pest genetics and ecology. Annu Rev Entomol 1998;43(1):701–726. 15012402 10.1146/annurev.ento.43.1.701

[12] Onstad DW. Insect resistance management: biology, economics, and prediction: Academic Press; 2013.

[13] Dively GP, Venugopal PD, Finkenbinder C. Field-evolved resistance in corn earworm to Cry proteins expressed by transgenic sweet corn. PLoS One 2016;11(12):e0169115 10.1371/journal.pone.0169115 28036388 PMC5201267

[14] Tabashnik BE, Carrière Y. Surge in insect resistance to transgenic crops and prospects for sustainability. Nat Biotechnol 2017;35(10):926 10.1038/nbt.3974 29020006

[15] Reisig DD, Huseth AS, Bacheler JS, Aghaee M, Braswell L, Burrack HJ, et al Long-term empirical and observational evidence of practical *Helicoverpa zea* resistance to cotton with pyramided Bt toxins. J Econ Entomol 2018;111(4):1824–1833. 10.1093/jee/toy106 29668958

[16] Bilbo TR, Reay-Jones FP, Reisig DD, Greene JK. Susceptibility of corn earworm (Lepidoptera: Noctuidae) to Cry1A. 105 and Cry2Ab2 in North and South Carolina. J Econ Entomol 2019 3 29; 10.1093/jee/toz062 30924858

[17] Archer T, Bynum E Jr. Corn earworm (Lepidoptera: Noctuidae) biology on food corn on the high plains. Environ Entomol 1994;23(2):343–348.

[18] Barber GW. Observations on the egg and newly hatched larva of the corn ear worm on corn silk. J Econ Entomol 1941;34(3):451–456.

[19] Caprio MA, Martinez JC, Porter PA, Bynum E. The impact of inter-kernel movement in the evolution of resistance to dual-toxin bt-corn varieties in *Helicoverpa zea* (Lepidoptera: Noctuidae). J Econ Entomol 2015;109(1):307–319. 10.1093/jee/tov295 26527792

[20] Dutton A, D Alessandro M, Romeis J, Bigler F. Assessing expression of Bt-toxin (Cry1Ab) in transgenic maize under different environmental conditions. IOBC WPRS BULLETIN 2004;27(3):49–56.

[21] US EPA. Biopesticides registration action document, *Bacillus thuringiensis* Cry1A.105 and Cry2Ab2 insecticidal proteins and the genetic material necessary for their production in corn. 2008; Available at: https://www3.epa.gov/pesticides/chem_search/reg_actions/registration/decision_PC-006514_30-Sep-10.pdf. Accessed 1/31, 2019.

[22] Chilcutt CF, Tabashnik BE. Contamination of refuges by *Bacillus thuringiensis* toxin genes from transgenic maize. Proc Natl Acad Sci U S A 2004 5 18;101(20):7526–7529. 10.1073/pnas.0400546101 15136739 PMC419639

[23] Yang F, Kerns DL, Head GP, Leonard BR, Levy R, Niu Y, et al A challenge for the seed mixture refuge strategy in Bt maize: impact of cross-pollination on an ear-feeding pest, corn earworm. PLoS One 2014;9(11):e112962 10.1371/journal.pone.0112962 25409442 PMC4237366

[24] Reisig DD, Kurtz R. Bt resistance implications for *Helicoverpa zea* (Lepidoptera: Noctuidae) insecticide resistance management in the United States. Environ Entomol 2018;47(6):1357–1364. 10.1093/ee/nvy142 30277503

[25] Hoy CW, Head GP, Hall FR. Spatial heterogeneity and insect adaptation to toxins. Annu Rev Entomol 1998;43(1):571–594. 15012398 10.1146/annurev.ento.43.1.571

[26] Carriere Y, Crowder DW, Tabashnik BE. Evolutionary ecology of insect adaptation to Bt crops. Evolutionary Applications 2010;3(5‐6):561–573. 10.1111/j.1752-4571.2010.00129.x 25567947 PMC3352503

[27] Yang F, Kerns DL, Brown S, Head GP, Huang F. Pollen contamination in seed mixture increases the dominance of resistance to Bt maize in *Spodoptera frugiperda* (Lepidoptera: Noctuidae). Pest Manag Sci 2017;73(11):2379–2385. 10.1002/ps.4631 28580723

[28] Gore J, Adamczyk J Jr, Blanco C. Selective feeding of tobacco budworm and bollworm (Lepidoptera: Noctuidae) on meridic diet with different concentrations of *Bacillus thuringiensis* proteins. J Econ Entomol 2005;98(1):88–94. 10.1603/0022-0493-98.1.88 15765669

[29] Orpet RJ, Degain BA, Unnithan GC, Welch KL, Tabashnik BE, Carrière Y. Effects of dietary protein to carbohydrate ratio on Bt toxicity and fitness costs of resistance in *Helicoverpa zea*. Entomol Exp Appl 2015;156(1):28–36.

[30] Dowd PF. Biotic and abiotic factors limiting efficacy of Bt corn in indirectly reducing mycotoxin levels in commercial fields. J Econ Entomol 2001;94(5):1067–1074. 10.1603/0022-0493-94.5.1067 11681667

[31] Horner T, Dively G, Herbert D. Development, survival and fitness performance of *Helicoverpa zea* (Lepidoptera: Noctuidae) in MON810 Bt field corn. J Econ Entomol 2003;96(3):914–924. 10.1603/0022-0493-96.3.914 12852636

[32] Neunzig H. Biology of the tobacco budworm and the corn earworm in North Carolina. NC Agric Exp Stn Tech Bull 1969;196.

[33] Wiseman BR, McMillian WW. Response of instars of the corn earworm *Heliothis zea* (Lepidoptera: Noctuidae), to two susceptible sweet corn hybrids. Ga Entomol Soc J 1973.

[34] Wiseman B, Widstrom N, McMillian W. Movement of corn earworm larvae on ears of resistant and susceptible corns. Environ Entomol 1978;7(5):777–779.

[35] Wiseman B, Widstrom N, McMillian W. Influence of corn silks on corn earworm feeding response. Fla Entomol 1981:395–399.

[36] Adamczyk J Jr, Adams L, Hardee D. Field efficacy and seasonal expression profiles for terminal leaves of single and double *Bacillus thuringiensis* toxin cotton genotypes. J Econ Entomol 2001;94(6):1589–1593. 10.1603/0022-0493-94.6.1589 11777069

[37] Nguyen HT, Jehle JA. Quantative analysis of the seasonal and tissue-specific expression of Cry1Ab in transgenic maize MON810. J Plant Dis Protec 2007;114(2):82–87.

[38] Székács A, Lauber É, Juracsek J, Darvas B. Cry1Ab toxin production of MON 810 transgenic maize. Environ Toxicol Chem 2010;29(1):182–190. 10.1002/etc.5 20821434

[39] Tian J, Collins HL, Romeis J, Naranjo SE, Hellmich RL, Shelton AM. Using field-evolved resistance to Cry1F maize in a lepidopteran pest to demonstrate no adverse effects of Cry1F on one of its major predators. Transgenic Res 2012;21(6):1303–1310. 10.1007/s11248-012-9604-4 22373893 PMC3505541

[40] Hung TP, Truong LV, Binh ND, Frutos R, Quiquampoix H, Staunton S. Persistence of detectable insecticidal proteins from *Bacillus thuringiensis* (Cry) and toxicity after adsorption on contrasting soils. Environ Poll 2016;208:318–325. 10.1016/j.envpol.2015.09.046 26549751

[41] Svobodová Z, Shu Y, Skoková Habuštová O, Romeis J, Meissle M. Stacked Bt maize and arthropod predators: exposure to insecticidal Cry proteins and potential hazards. Proc R Soc Lond B Biol Sci 2017;284(1859):20170440. 10.1098/rspb.2017.0440 PMC5543214 28724730

[42] Bradford MM. A rapid and sensitive method for the quantitation of microgram quantities of protein utilizing the principle of protein-dye binding. Anal Biochem 1976;72(1–2):248–254. 942051 10.1016/0003-2697(76)90527-3

[43] SAS Institute. User's manual, version 9.3: SAS Institute; 2011.

[44] Hurvich CM, Tsai C. Regression and time series model selection in small samples. Biometrika 1989;76(2):297–307.

[45] Kenward MG, Roger JH. Small sample inference for fixed effects from restricted maximum likelihood. Biometrics 1997:983–997. 9333350

[46] Tukey JW. The problem of multiple comparisons. Department of Statistics, Princeton University; 1953.

[47] Zalucki M, Furlong M. Behavior as a mechanism of insecticide resistance: evaluation of the evidence. Curr Opin Insect Sci 2017;21:19–25. 10.1016/j.cois.2017.05.006 28822484

[48] Bassetti P, Westgate ME. Water deficit affects receptivity of maize silks. Crop Sci 1993;33(2):279–282.

[49] Steckel S, Stewart S, Tindall K. Effects of Japanese beetle (Coleoptera: Scarabaeidae) and silk clipping in field corn. J Econ Entomol 2013;106(5):2048–2054. 10.1603/ec13042 24224246

[50] Ali A, Young S. Activity of *Bacillus thuringiensis* Berliner against different ages and stages of Helicoverpa zea (Lepidoptera: Noctuidae) on cotton. J Entomol Sci 1996;31(1):1–8.

[51] Cooper D. The application of a model to achieve predicted mortality in a field trial using *Bacillus thuringiensis* to control *Heliothis punctiger*. Entomol Exp Appl 1984;36(3):253–259.

[52] Bird LJ, Akhurst RJ. Variation in susceptibility of *Helicoverpa armigera* (Hübner) and *Helicoverpa punctigera* (Wallengren)(Lepidoptera: Noctuidae) in Australia to two *Bacillus thuringiensis* toxins. J Invertebr Pathol 2007;94(2):84–94. 10.1016/j.jip.2006.08.005 17049552

[53] Dulmage HT, Graham HM, Martinez E. Interactions between the tobacco budworm, *Heliothis virescens*, and the δ-endotoxin produced by the HD-1 isolate of *Bacillus thuringiensis* var. kurstaki: relationship between length of exposure to the toxin and survival. J Invertebr Pathol 1978;32(1):40–50.

[54] Luong TT, Zalucki MP, Perkins LE, Downes SJ. Feeding behaviour and survival of *Bacillus thuringiensis*‐resistant and *Bacillus thuringiensis*‐susceptible larvae of *Helicoverpa armigera* (Lepidoptera: Noctuidae) exposed to a diet with *Bacillus thuringiensis* toxin. Austral Entomol 2018;57(1):1–8.

[55] Deans CA, Behmer ST, Tessnow AE, Tamez-Guerra P, Pusztai-Carey M, Sword GA. Nutrition affects insect susceptibility to Bt toxins. Sci Rep 2017;7:39705 10.1038/srep39705 28045087 PMC5206677

[56] Deans CA, Sword GA, Behmer ST. Revisiting macronutrient regulation in the polyphagous herbivore *Helicoverpa zea* (Lepidoptera: Noctuidae): New insights via nutritional geometry. J Insect Physiol 2015;81:21–27. 10.1016/j.jinsphys.2015.06.015 26141409

[57] Deans CA, Sword GA, Lenhart PA, Burkness E, Hutchison WD, Behmer ST. Quantifying plant soluble protein and digestible carbohydrate content, using corn (*Zea mays*) as an exemplar. J Vis Exp 2018(138):e58164. 10.3791/58164 PMC6126640 30124669

[58] Behmer ST, Simpson SJ, Raubenheimer D. Herbivore foraging in chemically heterogeneous environments: nutrients and secondary metabolites. Ecol 2002;83(9):2489–2501.

[59] Joyner K, Gould F. Developmental consequences of cannibalism in *Heliothis zea* (Lepidoptera: Noctuidae). Ann Entomol Soc Am 1985;78(1):24–28.

[60] Horner T, Dively G. Effect of MON810 Bt field corn on *Helicoverpa zea* (Lepidoptera: Noctuidae) cannibalism and its implications to resistance development. J Econ Entomol 2003;96(3):931–934. 10.1603/0022-0493-96.3.931 12852638

[61] Chilcutt CF. Cannibalism of *Helicoverpa zea* (Lepidoptera: Noctuidae) from *Bacillus thuringiensis* (Bt) transgenic corn versus non-Bt corn. J Econ Entomol 2006;99(3):728–732. 10.1603/0022-0493-99.3.728 16813305

[62] Storer NP, Van Duyn JW, Kennedy GG. Life history traits of *Helicoverpa zea* (Lepidoptera: Noctuidae) on non-Bt and Bt transgenic corn hybrids in eastern North Carolina. J Econ Entomol 2001;94(5):1268–1279. 10.1603/0022-0493-94.5.1268 11681693

[63] Reisig DD, Reay-Jones FP. Inhibition of *Helicoverpa zea* (Lepidoptera: Noctuidae) growth by transgenic corn expressing Bt toxins and development of resistance to Cry1Ab. Environ Entomol 2015;44(4):1275–1285. 10.1093/ee/nvv076 26314074

[64] US EPA. Biopesticides registration action document, Bacillus thuringiensis Cry1F corn. 2005; Available at: https://archive.epa.gov/pesticides/biopesticides/web/pdf/brad_006481.pdf. Accessed 1/31, 2019.

[65] Buntin GD. Corn expressing Cry1Ab or Cry1F endotoxin for fall armyworm and corn earworm (Lepidoptera: Noctuidae) management in field corn for grain production. Fla Entomol 2008;91(4):523–531.

[66] Bilbo TR, Reay-Jones FP, Reisig DD, Musser FR, Greene JK. Effects of Bt corn on the development and fecundity of corn earworm (Lepidoptera: Noctuidae). J Econ Entomol 2018;111(5):2233–2241. 10.1093/jee/toy203 29986034

[67] US EPA. *Bacillus thuringiensis* subspecies Cry1F protein and the genetic material necessary for its production (plasmid onsert PHI 8999) in corn fact sheet. 2001; Available at: https://archive.epa.gov/pesticides/biopesticides/web/html/factsheet_006481.html. Accessed 4/23, 2019.

[68] Onstad DW, Crespo AL, Pan Z, Crain PR, Thompson SD, Pilcher CD, et al Blended refuge and insect resistance management for insecticidal corn. Environ Entomol 2017;47(1):210–219. 10.1093/ee/nvx172 PMC5850660 29220481

[69] FIFRA Scientific Advisor Panel, Report. Resistance of Lepidopteran pests to *Bacillus thuringiensis* (Bt) plant incorporated protectants (PIPs) in the United States. 2018; Available at: https://www.regulations.gov/contentStreamer?documentId=EPA-HQ-OPP-2017-0617-0078&contentType=pdf. Accessed 4/3, 2019.

[70] Kaur G, Guo J, Brown S, Head GP, Price PA, Paula-Moraes S, et al Field-evolved resistance of *Helicoverpa zea* (Boddie) to transgenic maize expressing pyramided Cry1A. 105/Cry2Ab2 proteins in northeast Louisiana, the United States. J Invertebr Pathol 2019;163:11–20. 10.1016/j.jip.2019.02.007 30825480

[71] Burkness EC, Dively G, Patton T, Morey AC, Hutchison WD. Novel Vip3A *Bacillus thuringiensis* (Bt) maize approaches high-dose efficacy against *Helicoverpa zea* (Lepidoptera: Noctuidae) under field conditions: Implications for resistance management. GM crops 2010;1(5):337–343. 10.4161/gmcr.1.5.14765 21844691

[72] Yang F, Kerns DL, Leonard BR, Oyediran I, Burd T, Niu Y, et al Performance of Agrisure® Viptera™ 3111 corn against *Helicoverpa zea* (Lepidoptera: Noctuidae) in seed mixed plantings. Crop Prot 2015;69:77–82.

[73] Yang F, González JCS, Williams J, Cook DC, Gilreath RT, Kerns DL. Occurrence and ear damage of *Helicoverpa zea* on transgenic *Bacillus thuringiensis* maize in the field in Texas, U.S., and its susceptibility to Vip3A protein. Toxins 2019;11:102. 10.3390/toxins11020102 PMC6416581 30744120

